# Surface Topography Induces and Orients Nematic Swarms of Active Filaments: Considerations for Lab-On-A-Chip Devices

**DOI:** 10.1021/acsanm.4c02020

**Published:** 2024-05-08

**Authors:** Joseph M. Barakat, Kevin J. Modica, Le Lu, Stephanie Anujarerat, Kyu Hwan Choi, Sho C. Takatori

**Affiliations:** Department of Chemical Engineering, University of California, Santa Barbara, Santa Barbara, California 93106, United States

**Keywords:** active matter, surface topography, collective motion, active nematics, actin filaments

## Abstract

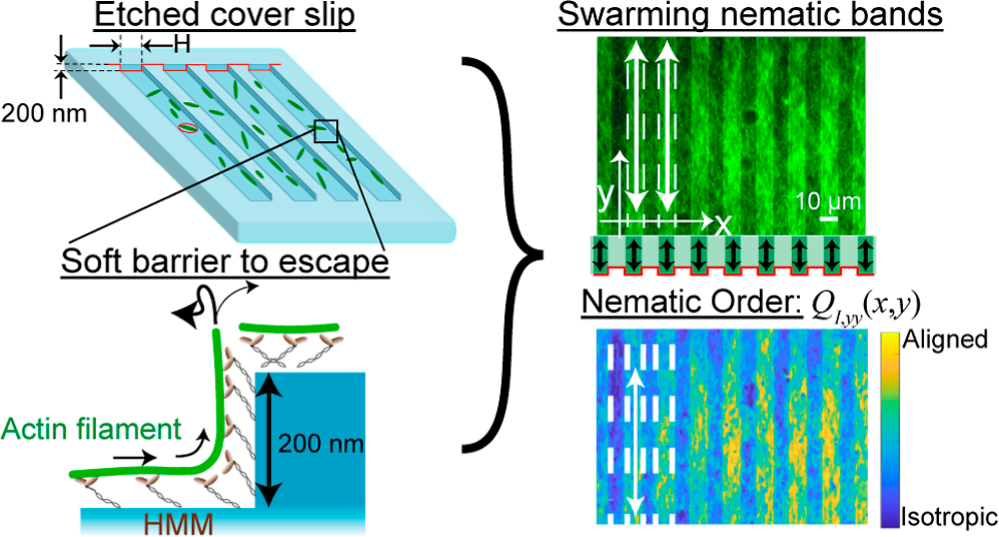

Surface-bound molecular motors can drive the collective motion of cytoskeletal filaments in the form of nematic bands and polar flocks in reconstituted gliding assays. Although these “swarming transitions” are an emergent property of active filament collisions, they can be controlled and guided by tuning the surface chemistry or topography of the substrate. To date, the impact of surface topography on collective motion in active nematics is only partially understood, with most experimental studies focusing on the escape of a single filament from etched channels. Since the late 1990s, significant progress has been made to utilize the nonequilibrium properties of active filaments and create a range of functional nanodevices relevant to biosensing and parallel computation; however, the complexity of these swarming transitions presents a challenge when attempting to increase filament surface concentrations. In this work, we etch shallow, linear trenches into glass substrates to induce the formation of swarming nematic bands and investigate the mechanisms by which surface topography regulates the two-dimensional (2D) collective motion of driven filamentous actin (F-actin). We demonstrate that nematic swarms only appear at intermediate trench spacings and vanish if the trenches are made too narrow, wide, or tortuous. To rationalize these results, we simulate the F-actin as self-propelled, semiflexible chains subject to a soft, spatially modulated potential that encodes the energetic cost of bending a filament along the edge of a trench. In our model, we hypothesize that an individual filament experiences a penalty when its projected end-to-end distance is smaller than the trench spacing (“bending and turning”). However, chains that span the channel width glide above the trenches in a force- and torque-free manner (“crowd-surfing”). Our simulations demonstrate that collections of filaments form nematic bands only at intermediate trench spacings, consistent with our experimental findings.

## 1 Introduction

Filamentous active matter, including filamentous actin (F-actin) and microtubules, are essential components of the cytoskeleton.^1,2^ The seminal work by Kron and Spudich established the F-actin gliding assay for visualizing single filaments driven by surface-bound myosin motors.^3^ Active filaments exhibit collective dynamics that hold promise for the development of miniaturized, multifunctional lab-on-a-chip (LOC) devices. These devices could potentially utilize orchestrated groups of filaments for tasks such as analyte sensing, parallel computation, and targeted cargo delivery.^4,5^ While significant research efforts have focused on exploiting individual filaments in nanodevices, a growing area of interest lies in harnessing the emergent properties that arise from collective filament behavior. Researchers have already utilized the gliding assay to demonstrate the emergence of polar flocks and nematic swarms in collections of F-actin at moderate surface densities.^6−10^ Theory and agent-based simulation have demonstrated that the emergence of these “swarming transitions” are determined by the symmetry of the interaction between colliding filaments.^6,8,11^ If the pairwise collision of two filaments has nematic symmetry, the system will form nematic bands at a high density; however, if the pairwise collision preferentially aligns the filaments to point the same direction, the system can form polar flocks. While experiments have shown the presence of both nematic swarms and polar flocks, excluded-volume interactions alone only exhibit nematic symmetry upon the inelastic collision of two filaments. While the mechanics of the isotropic to polar or nematic transition is well understood for individual filaments, controlling the onset and evolution of filamentous swarms and flocks remains challenging, as these structures are inherently nonequilibrium and localized in space and time.^12,13^ Furthermore, the collective patterns observed in dry active systems, like gliding F-actin, showed a density-dependent polar transition, whereas other active nematics in wet systems exhibited dynamics driven by fluid-mediated hydrodynamic interactions.^14,15^

Researchers have proposed different methods to control the collective behavior of two-dimensional (2D) active matter.^16^ Strategies include light-activated force generation,^17−19^ thermotropic liquid crystals,^20−22^ boundary confinement,^23−25^ surface chemistry patterning,^26−30^ and surface topography.^31−41^ The latter has been shown to direct the motion of individual filaments in dilute systems of kinesin-driven microtubules^31−34^ and myosin-driven F-actin.^36−41^ By comparison, relatively few experimental studies have examined the effect of surface topography on collections of filaments at high surface densities.^15,42,43^

Theory and simulation have demonstrated various methods to control swarming transitions in 2D active nematics, including boundary effects.^12,44−49^ In the presence of hydrodynamic interactions, active nematics confined between two parallel walls undergoes a transition between active turbulence, an ordered vortex lattice with dynamically structured disclinations, and coherent flow when the channel spacing and active stress decrease relative to the system’s effective Frank elastic constant.^50^ Similar results have been found for active nematics in a disk and annulus, with the boundary curvature allowing circulating and corotating states.^51,52^ The behavior of these wet systems is characterized by the presence and motion of topological defects in the director field. In dry systems, it remains unclear how confinement and surface topography influence the order–disorder transitions between isotropic motion, polar flocks, and nematic swarms in *collections* of filaments, precluding the possibility of precise control.

In this work, we study the effect of surface topography on the collections of dry active nematics. We manipulate surface topography by introducing periodic shallow trenches in myosin-coated glass substrates to trigger the formation of nematic swarms within F-actin systems. These trenches are shallow enough that the F-actin is able to bend out of plane and escape the trenches to glide along the surrounding hills or re-enter the trenches after gliding on the hills.

In low-density systems, we find that individual F-actin exhibits enrichment along the trench boundaries, consistent with previous studies.^36−41^ At higher F-actin densities, we observe the development of swarming nematic bands characterized by spatially modulated density and nematic order along the channels. Notably, the swarming nematic bands form only at intermediate trench spacing, and the nematic order is nonmonotonic with channel spacing. Narrow trenches suppress the formation of swarms altogether, whereas wide trenches result in uncorrelated collective motion. Similar suppression is observed for tortuous trenches compared to linear ones. Interestingly, for the intermediate channel spacing, swarms consistently manifest and align along the channels. These experimental findings suggest the existence of an optimal length scale for surface topography to effectively guide the collective motion of the filaments.

To rationalize our experimental findings, we developed a computational model of 2D self-propelled filaments subject to bending forces and torques along periodic intervals in one direction. Filaments spanning more than one periodic cell are assumed to glide freely without bending, whereas collapsed filaments are forced to bend and turn. This model is then implemented in 2D Brownian dynamics simulations of a collection of self-propelled, mutually interacting filaments, which demonstrate significant nematic ordering and swarming when the filament length is comparable to the periodic repeat distance. Our model and simulations are consistent with the experimental observation that the “optimal” trench spacing is comparable to the run length and persistence length of F-actin. Taken together, our experiments and simulations suggest that the relationship between surface topography and self-propulsion is strongly coupled and that precise tuning of topographical surfaces is necessary to promote and direct swarming behavior in filamentous active matter.

## 2 Materials and Methods

### 2.1 Experimental Details of F-Actin Gliding Assay on Microfabricated Etched Topographies

We microfabricated etched features on a 24 × 40 mm, 170 μm thickness borosilicate coverslip (Azer Scientific). We used the Heidelberg Maskless Aligner, high-speed direct-write photolithography equipment that is available at the UCSB Nanofabrication Facility for rapid prototyping and high-throughput of etched patterns. We developed a photolithographic mask on the substrate with a specified pattern, followed by anisotropic plasma dry etching with CF_4_/CHF_3_ gases.^53^ Using this technique, we created topographic patterns on the coverslip because the photoresist will mask select regions from dry etching.^53^ Once the topographical substrates were created, we followed the existing methods for actin-gliding assays.^6,54^ We purified heavy meromyosin (HMM) motor proteins and globular actin monomers (G-actin) from rabbit skeletal muscle.^55−57^

We coated the etched coverslip with a thin layer of trichloromethylsilane and created an observation chamber on the coated substrate using a 5 mm thick polydimethylsiloxane (PDMS, Sylgard 184, Dow) block with a 6 mm hole. We deposited a F-buffer containing HMM into the observation chamber to allow the HMM to stick on the substrate. The F-buffer composition is 50 mM Tris pH 7.5, 2 mM MgCl_2_, 0.2 mM CaCl_2_, 25 mM KCl, 0.5 mM adenosine 5′-triphosphate (ATP), and 1 mM dithiothreitol (DTT). Separately, 10 μM G-actin and 1 μM phalloidin-647 (Alexa Fluor Plus 647 Phalloidin, Invitrogen) were added to the F-buffer and incubated for 45 min. The F-actin suspension was pipetted into the observation cell at the desired density. We allowed the F-actin to sediment for 30 min and added ATP at a specified density, following previous actin-gliding assays.^3,6^ Activity persisted for at least 30 min after ATP addition. An ATP regeneration system was not necessary for our system.

All imaging was carried out on an inverted Nikon Ti2-Eclipse microscope (Nikon Instruments) using an oil-immersion objective (Plan Apochromat VC 100×, numerical aperture 1.4). A Lumencor SpectraX Multi-Line LED light source was used for excitation (Lumencor, Inc.). Fluorescent light was spectrally filtered with an emission filter (680/42; Semrock, IDEX Health and Science) and imaged on a Photometrics Prime 95 CMOS Camera (Teledyne Photometrics).

### 2.2 Brownian Dynamics Simulations of Active Filaments

An active filament is represented as a chain of 21 spherical particles with a diameter of σ connected by harmonic spring forces between adjacent pairs and bond-angle forces between connected triplets. The position **r**_*i*,*j*_(*t*) of the *j*th particle on the *i*th chain is advanced via the Langevin equation
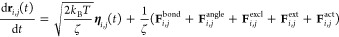
1where ζ is the particle drag coefficient and **η**_*i*,*j*_ is a white-noise source. In this model, each particle experiences forces due to bond pairs, angular triplets, excluded-volume interactions, external fields, and active propulsion, each of which is discussed further below.

The bond and angle forces, **F**_*i*,*j*_^bond^ and **F**_*i*,*j*_^angle^, between particles on the *same* chain are defined as follows. For two bonded particles separated by a distance *r*, a harmonic spring potential pulls them to an equilibrium separation equal to their diameter
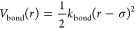
2

Similarly for a group of three linearly bonded particles forming an angle θ, an angle potential pulls them into parallel alignment
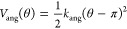
3

Here, *k*_bond_ and *k*_ang_ denote the Hookean spring stiffnesses for these potentials. To simulate semiflexible filaments, we choose a large bond stiffness, *k*_bond_ = 50*k*_B_*T*, and an angle stiffness that gives a persistence length, *L*, equal to the contour length, *L*_c_ = 21σ, and *k*_ang_ = 21*k*_B_*T*. The bond and angle forces are then obtained from a virtual work argument
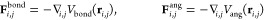
4where ∇_*i*,*j*_ denotes the gradient with respect to the position of the *j*th particle on the *i*th chain.

Any two particles not directly bonded to each other interact with each other via the following soft excluded-volume interaction
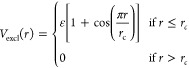
5where ε = 10*k*_B_*T* is the energy-well depth and *r*_c_ = 1.1σ is the interaction radius. The excluded-volume force is then computed via the gradient
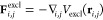
6

To simulate an externally imposed, soft confinement with periodic repeat distance 2*H*, we use a continuous approximation of the square-wave potential
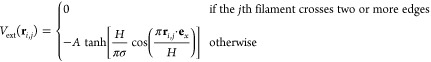
7where *A* is the potential amplitude. Here, an “edge” denotes an inflection point in the potential landscape where a “hill” meets a “valley”. This external field is defined such that a filament crossing multiple edges feels no force or torque. The number of edges crossed, *N*_*i*,cross_ by the *i*th chain with its tail at *x*_*i*,1_ and head at *x*_*i*,N_ is found via the following algorithm
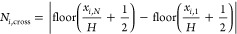
8

The amplitude, *A*, of the external field is calibrated such that the work required for a filament to escape a trench is 2*AL*_c_/σ = 42*k*_B_*T*. The associated force on the *j*th particle on the *i*th chain is
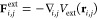
9

Finally, active propulsion is modeled by imposing a constant force, *F*_act_, on each particle along the tangent of the filament contour^58^
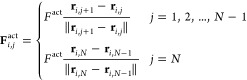
10where *N* = 21 is the number of particles per chain. The strength of the active propulsion force is set to be half of the value of the maximum external-potential force. Hence, a single particle on the chain will not easily overcome the energy barrier, but multiple particles directed together can push the chain out of a local potential well.

A system of filaments with number density ρ was initialized in a periodic box of width *L*_*x*_ and length *L*_*y*_. The box width was determined from an integer multiple of the periodic repeat spacing of the external potential, *L*_*x*_ = 2H*p. The periodicity, *p*, and number of filaments were adjusted such that the box width *L*_*x*_ > 20*L*_c_ and the box length *L*_*y*_ > 50*L*_c_, to avoid finite-size effects from the periodic boundary conditions.

After calibrating our parameters, we ran simulations to determine the impact of interfilament interactions on ordering and alignment. All simulations were performed at the number density ρ = 3/*L*_c_^2^. We calculated the density-weighted nematic order tensor, **Q**, by finding the unit orientation vector **q** for all filaments at all timesteps (resulting in *N*_samp_ number of samples), forming a symmetric and traceless dyad, and then taking the ensemble average. The ensemble-averaged nematic order tensor was found for three independent simulations, and the standard deviation of the average values was computed to find the error of the mean.

## 3 Results and Discussion

### 3.1 Surface Topography Directs the Motion of Individual Filaments and Dense Swarms

**Figure 1:**
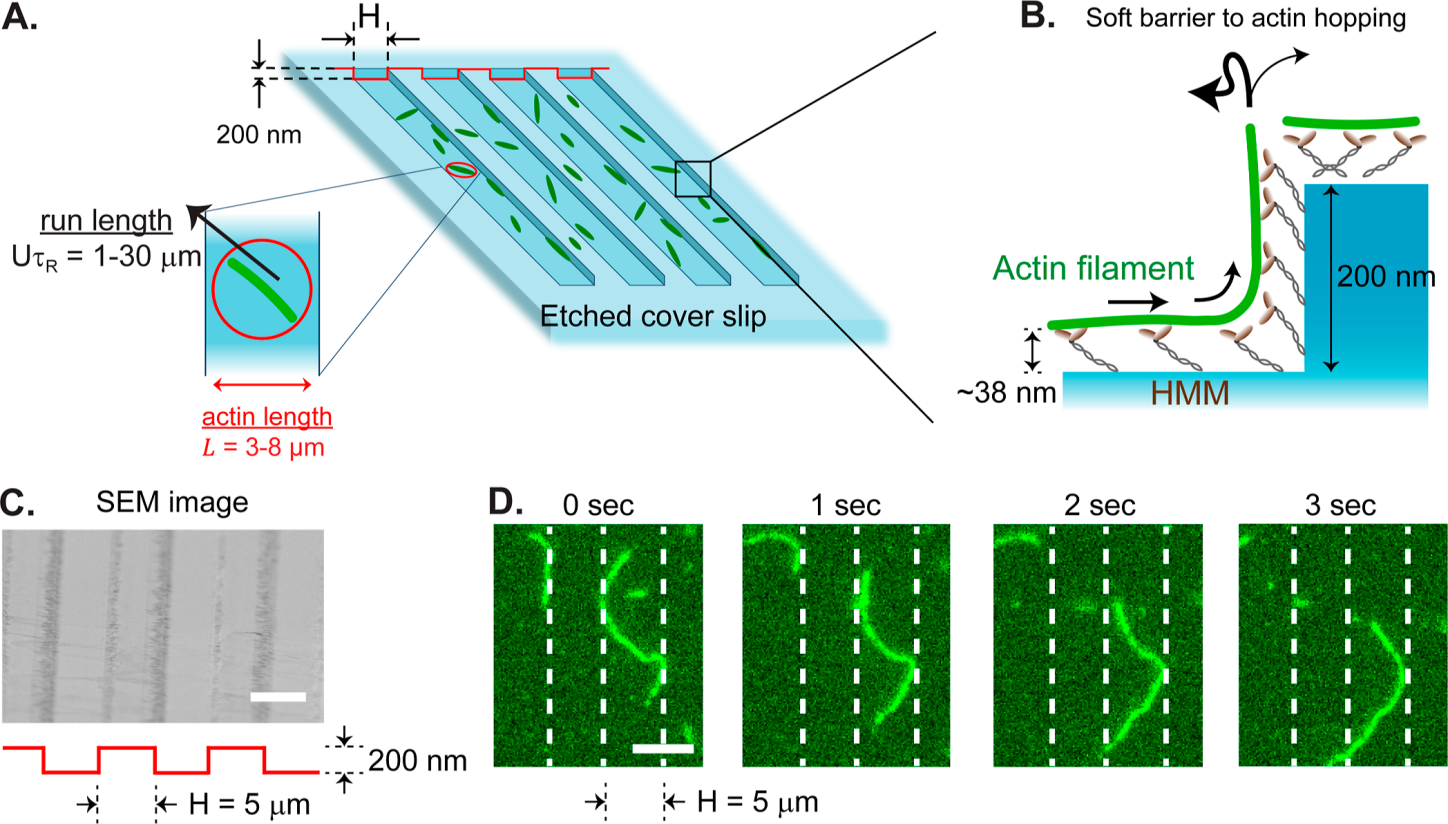
Etched surface topographies are used to control the motion and swarming transitions of filamentous actin (F-actin) propelled by surface-bound heavy meromyosin (HMM) motors. (A) Schematic of parallel trenches with shallow valleys and variable spacing, *H*. (B) Etched substrates of depth ≈ 200 nm impose a soft confinement for F-actin inside the trenches. (C) Scanning electron microscopy image of the etched substrates with parallel trenches (5 μm channels shown here). (D) Individual actin filaments bounce off the walls due to a soft confinement potential imposed by the trench edges. All scale bars are 5 μm.

Figure 1A,B depicts our etched coverslips coated with molecular motors to direct the motion of F-actin in a gliding assay (for details, see the Materials and Methods section). The etched trenches have a fixed depth ≈ 200 nm, as verified by scanning electron microscopy (Figure 1C), and a periodic repeat spacing, *H*, that can be controlled. Figure 1D depicts measurements for single filaments with a trench spacing *H* = 5 μm. Upon activating the system via addition of ATP, we observed individual F-actin collide into the trench boundaries and reorient their motion within the confines of the trenches. Since F-actin glides at an average height of ≈38 nm above the substrate,^59^ we hypothesized that the filaments experience a soft barrier to bending at the sharp edge of the trench (Figure 1B) similar to the confinement observed for kinesin-propelled microtubules in microfluidic channels with deep trenches.^32−34^ The flexural modulus of phalloidin-stabilized F-actin^16^ is *EI* ≈ 20 *k*_B_*T*·μm and so the work to bend a filament segment into a quarter-arc of radius ≈ 200 nm is of order 50 *k*_B_*T*.^60^ Therefore, although thermal Brownian forces are unlikely to drive the filaments out of the trenches, active forces generated by the myosin motors can overcome the bending penalty posed by the trench edge.

**Figure 2:**
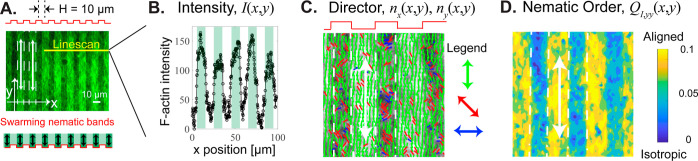
Parallel trenches induce and guide nematic bands of F-actin. (A) Image of gliding assay showing swarming nematic bands of F-actin moving up and down along the periodic parallel channels (outlined in white dashed lines). (B) Line scan of local intensity, *I*(**r**) shows periodic, four-fold enrichment of F-actin. (C) The unit director field, **n**(**r**), is strongly aligned with the direction of the trenches. (D) Contours of the *yy* component of the nematic order tensor, **Q**_*I*_(**r**), as defined by eq 11.

At larger F-actin surface densities, we observed the spontaneous formation of swarming nematic bands along the trenches that repeated every periodic spacing (Figure 2A; see Supplementary Movie 1). These robust nematic bands spanned hundreds of micrometers and persisted over tens of minutes until ATP depletion. Filaments continuously enter and leave the bands while maintaining an enriched density within the trenches (line scan in Figure 2B shows approximately four-fold enrichment). The nematic bands were similar in structure to those observed in F-actin gliding assays on planar substrates;^6^ here, we demonstrated our ability to control the alignment of nematic filaments using surface topography.

To confirm the development of spatially modulated order within the system, we computed the intensity-weighted nematic order tensor^61^
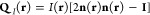
11as a function of position , where *I*(**r**) is the normalized scalar intensity and **n**(**r**) is the unit director (Figure 2B,C). The concentration of filaments is not uniform in space; we use the intensity as a proxy for actin density when sampling the F-actin nematic order. Contour plots of the *yy* tensor component (parallel to the trenches) confirm the formation of ordered, nematic bands within the trenches (Figure 2D). The average degree of order for a particular experiment is quantified by the areal average of the order tensor
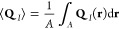
12where d**r** = d*x* d*y* and *A* denotes the two-dimensional area in the field of view. Equation 12 is used to compare the degree of ordering across different experiments.

### 3.2 Two-Dimensional (2D) Model of Topography-Directed Active Filaments

**Figure 3:**
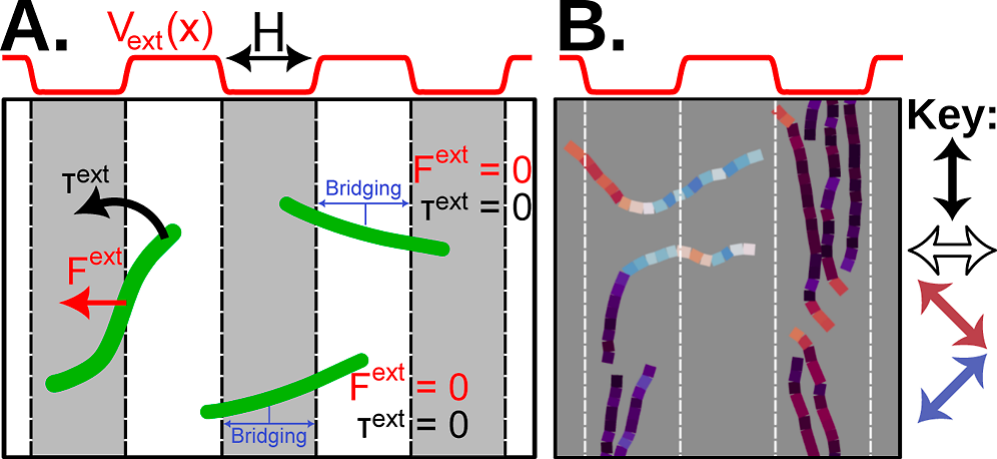
Actin gliding captured in simulations using a 2D potential model. (A) Schematic demonstrating our model of topographical confinement using an external field. Active filaments are confined and turned by the external field only if the actin is not bridging the channel. If a filament bridges between two hills or valleys (represented as potential energy minima and maxima), the filament ignores the energy field and glides across channels under zero force and torque, **F**^ext^ = **0** and *T*^ext^ = 0. (B) Simulation snapshot of active filaments moving under the influence of the potential field. Filaments are colored by their local nematic order along the contour.

In order to develop a mechanistic understanding of topography-driven spatial enrichment and nematic ordering of active filaments, we model the topographical surface as a spatially modulated potential in the *x*–*y* plane. This 2D representation offers favorable computational efficiency when applied to a large number of filaments. Our potential model, as illustrated in Figure 3, derives from the argument that filaments experience local bending forces and torques near the edge of a trench. Under the action of a square-wave potential with periodicity in the *x*-direction, a single filament is forced toward the potential minima and torqued to align along the *y*-direction (Figure 3A). Groups of mutually exclusive filaments under such forces and torques will coordinate their motion to run transverse to the potential gradients. We call this type of behavior “bending and turning”.

However, when an active filament is longer than the periodic spacing, a single filament has to sharply bend at multiple locations, which is energetically unfavorable. In this scenario, we expect the filament to detach from the motors coating the depressed regions of the topographical surface rather than bend at multiple points. Active propulsion is then solely generated by motors on the elevated surfaces. This latter type of behavior is called “crowd-surfing” since the filament glides above the trenches without bending. In order to model this behavior, we switched off the potential whenever the end-to-end distance of a filament spans multiple periodic cells (Figure 3A). This phenomenological rule is inspired by experimental observations of F-actin that predominantly glide in a straight path on the tops of the trenches for the small-wavelength trenches, unaffected by the undulations of the substrate.

We implemented this potential model in 2D Brownian Dynamics (BD) simulations of semiflexible filaments of persistence length *L* = 10 μm and contour length *L*_c_ = 10 μm subject to thermal, active, and potential-driven forces as well as pairwise excluded-body forces. Details of our simulation methodology can be found in the Materials and Methods section; a sample simulation is shown in Figure 3B. Simulation parameters were first calibrated for a dilute system, for which the excluded-body forces are disabled, before increasing the surface concentration to determine the impact of interfilament interactions on ordering and alignment. To compare against our experimental results on a qualitative basis, we calculated the ensemble-averaged, density-weighted nematic order tensor
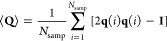
13where **q**(*i*) is unit orientation vector (directed along the propulsion axis) for the *i*th sampled filament at a particular time step, *N*_samp_ is the total number of samples (filaments and timesteps), and ⟨···⟩ denotes an ensemble average over all simulated trajectories. The latter should not be confused with the areal average in eq 12, which is intended for the limited sampling window observed in our experiments.

### 3.3 Swarm Suppression by Varying the Periodic Repeat Spacing

**Figure 4:**
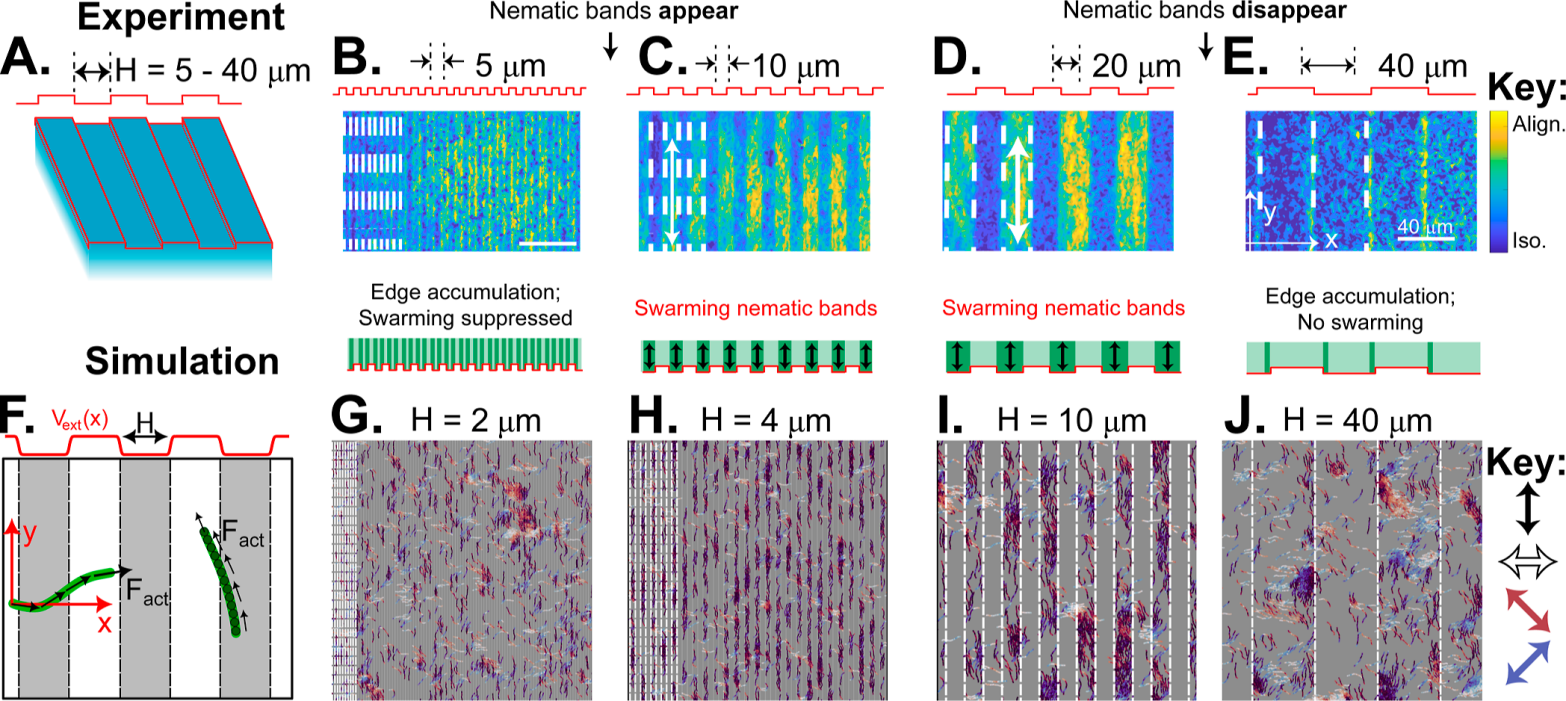
Nematic bands appear at intermediate spacing, *H*, but not at large and small spacing. (A) Schematic of our experiments on etched coverslips. (B–D) Contours of the *yy* component of the nematic order tensor, **Q**_*I*_(**r**). All experiments were conducted at the same ATP concentration, 0.1 mM. (B) At small spacing, *H* = 5 μm, we do not observe swarming nematic bands. (C,D) At intermediate spacing, *H* = 10–20 μm, nematic bands form along the channels with significant actin enrichment. (E) At large spacing, *H* = 40 μm, we see an isotropic distribution of actin filaments, with a small accumulation at the periodic edges. (F) Schematic of the simulation box with a periodically modulated potential. (G–J) BD simulations varying the potential width corroborate our experimental observations.

In our experiments, we varied the trench spacing across *H* = 5–40 μm while keeping the F-actin surface density and ATP concentration fixed (Figure 4). Interestingly, we found that the large (*H* = 40 μm) and small (*H* = 5 μm) trench spacings do not produce nematic bands (see Supplementary Movies 2 and 3 for videos of *H* = 5 μm and *H* = 40 μm, respectively). For a small spacing, *H* = 5 μm, we never observed nematic bands in any of our experiments (more than 30 replicates). For large spacing, *H* = 40 μm, we occasionally observed nematic bands that are similar to the bulk swarms in unconfined systems,^6,12^ but these bulk swarms were not correlated with the periodic trenches. In contrast, nematic bands formed consistently in the direction of the channels over intermediate trench spacings of *H* = 10–20 μm (Figure 4C,D). These intermediate spacings are similar to the characteristic length scale associated with the actin swarms observed previously on planar surfaces.^6^ Taken together, our data suggest that the nematic bands appear in our periodic channels when the spacing, *H*, is comparable to the orientation screening length of the swarms.

Using our 2D model, we also performed BD simulations of active filaments in a potential field of varying periodic repeat spacing *H* (Figure 4F–J and Supplementary Movies 4, 5, and 6). Similar to our experiments, our simulations indicated that swarms preferentially align along the potential wells at an intermediate spacing, *H* = 3–12 μm. The physical mechanism behind this preferential alignment at intermediate spacing is linked to an energetic competition between filament bending and adhesion to the substrate, as discussed previously.

**Figure 5:**
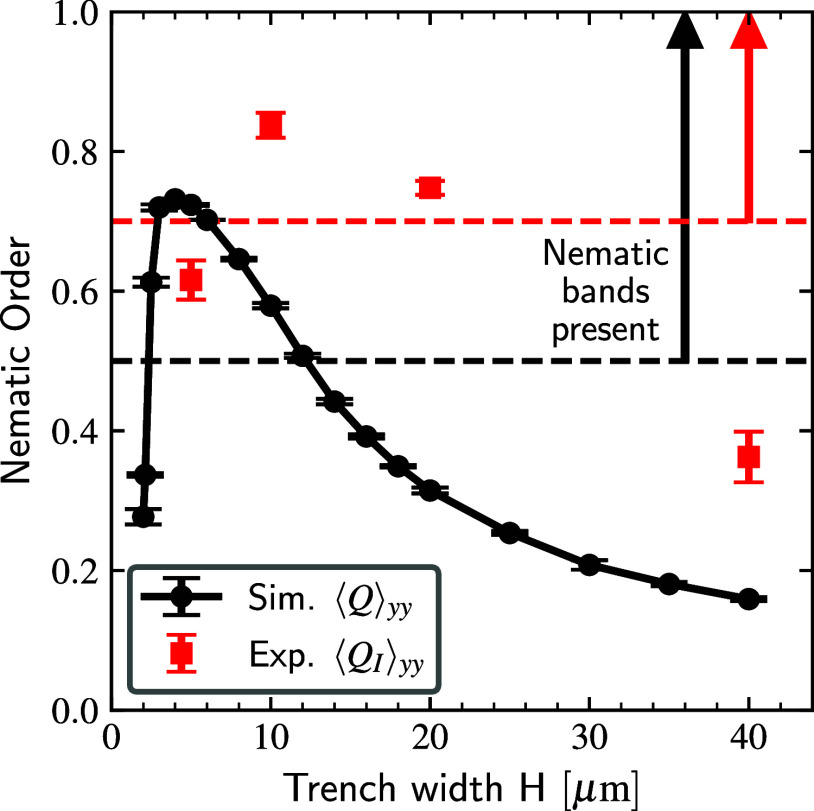
Experiments and simulations indicate that filament alignment is maximized at intermediate trench spacing. Simulated filaments have contour length *L*_c_ = 10 μm to roughly match the mean experimental actin length. The degree of alignment of filaments with the channel director is plotted as a function of trench width. Black circles are the density-weighted nematic order measured from simulations, ⟨**Q**⟩_*yy*_. Red squares are the intensity-weighted average nematic order from the experiments, . These metrics quantify the appearance of swarming nematic bands over an intermediate trench spacing, H*. The simulations underpredict (*H*_sim_^*^ ≈ 4 μm) the value of H* observed in the experiments (*H*_exp_^*^ ≈ 10 μm). Dashed lines in both figures are markers of visual observations of the appearance of nematic bands in simulation (black) and experiment (red).

To quantify the onset of swarming, we computed the average nematic order tensors ⟨**Q**_*I*_⟩ and ⟨**Q**⟩ from the experiments and simulations, respectively (cf. eqs 12 and 13). Since these tensors are weighted differently (by intensity in the experiments and by density in the simulations), the comparison between the two is intended to be qualitative rather than quantitative. The *yy* components of each are coplotted in Figure 5 as a function of the periodic repeat spacing (or trench width), *H*. Both the experiments and simulations indicate that order is maximized at an intermediate trench spacing, H*. At small trench spacings (*H* < H*), filaments align along boundaries but do not form collective nematic bands, resulting in low nematic order. As the spacing is increased (*H* ≈ H*), ordered nematic bands appear in both simulation and experiment. Actin propels along a director parallel to the trenches in bundles made from a large number of individual filaments. At large trench spacing (*H* > H*), the boundaries are far enough apart that orientational correlations decay by the time the filament reaches the center of the trenches, resulting in a loss of global order.

The simulations predict a value H* ≈ 4 μm that is smaller than the experimental observation, ≈10 μm, which we believe could be caused by polydispersity in the experimental actin contour lengths. Nevertheless, the qualitative agreement between the experiments and simulations indicate that the microscopic rule proposed in our simple model can explain the nonmonotonic dependence of nematic order with trench spacing. To recapitulate, the basic idea of this rule is that filaments will bend and turn to align with the edge of a trench only if their length does not span the entire trench width. A filament crossing one edge will start to turn until it reaches the second edge; this implies that most filaments will cross the second edge at an angle relative to the *x*-axis, provided their run length is sufficiently small. This explains why the optimal trench width for alignment as predicted in the simulation (≈4 μm) is smaller than the filament length (≈10 μm). More accurate predictions would likely require a (much more computationally intensive) 3D simulation of filament motion, explicitly resolving the spatial variation in depth (i.e., in the *z*-direction).

As discussed previously, we estimate the bending penalty for actin to be ≈50*k*_B_*T* at each of the trench corners. A filament that spans multiple channels can avoid this bending penalty by detaching from *N*_bound_ bound HMM motors. The precise dissociation energy between actin and myosin depends upon the experimental conditions (ionic strength, pH, presence of ATP, etc.), but we estimate from previously reported values for the dissociation constant that Δ*G*_dissoc_ < 10*k*_B_*T* per motor.^62^ Since HMM is a nonprocessive motor, actin is rapidly binding and unbinding with the surface-bound motor and stochastically sampling the competing bending and binding energies within the trenches. We model the crossover of the filament into the “crowd-surfing” state when the filament crosses two or more trench boundaries.

In experiments on planar gliding assays, collisions between F-actin have been shown to have a slight polar symmetry, leading to large density fluctuations and nematic bands or polar flocks depending on experimental conditions.^8^ In our model, interactions between filaments are purely steric with nematic symmetry. Generally, agent-based simulations and theories prescribe an additional alignment interaction to capture experimentally observed polar flocks, but here, we focus on the isotropic to nematic transition. Therefore, this model will not capture the formation of bulk polar flocks as seen in some experiments.

An important distinction between the experiments and simulations is the complete suppression of collective swarms at small trench spacings. In the simulations, by contrast, collective swarms are observed even when the periodic repeat spacing is small, but the directional motion of these swarms does not correlate with the applied potential field. Rather, the simulated swarms “surf” across the peaks and valleys of the potential landscape. We attribute the absence of swarming in the experiments at small trench spacings to additional physics not taken into account in the simulations. For instance, scission events at the trench boundaries produce successively smaller filaments and reduce their ability to orient with one another. Such events occur more frequently when the trench spacing is narrow, which could possibly explain the absence of swarms in the experiments. By contrast, in the simulations, the contour length of each filament is fixed, and collisions between filaments can give rise to spontaneous collective motion. Bending of the filaments into the third dimension is another feature that is present in the experiments but not in the simulations.

### 3.4 Impact of Propulsion Speed and Trench Tortuosity on Swarm Formation

**Figure 6:**
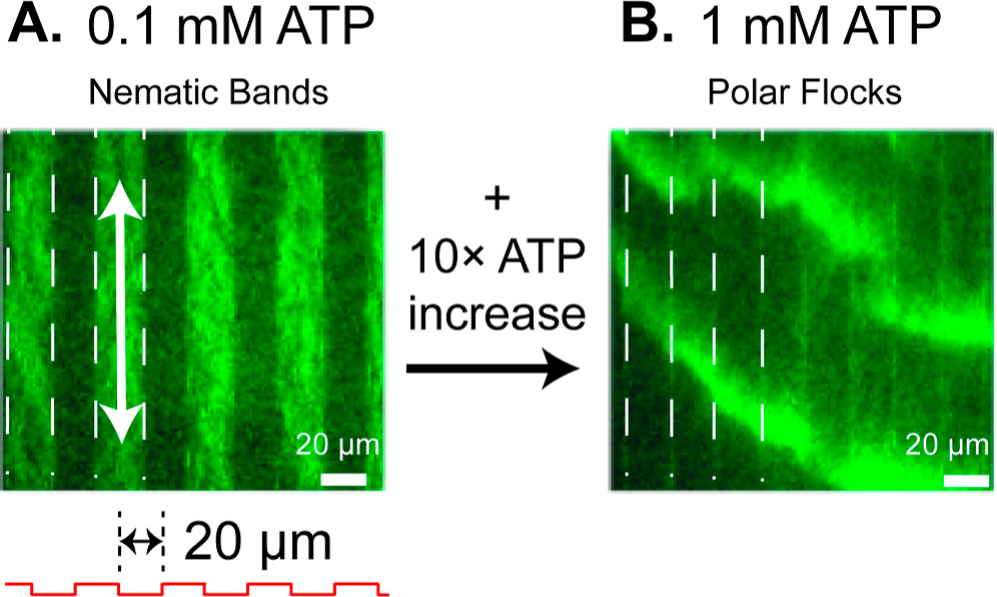
Topography-induced nematic bands only appear at intermediate propulsion speeds. (A) Nematic bands appear at intermediate ATP concentration, 0.1 mM. (B) Nematic bands vanish along the periodic trenches upon increasing ATP concentration to 1 mM. Polar flocks appear after 15 min, indicating that actin motion is unaffected by topography at high ATP concentration. These experiments were conducted with the same channel spacing, *H* = 20 μm.

In a separate set of experiments, we varied the ATP concentration to study the role of propulsion speed in nematic band formation (Figure 6). At ATP concentrations between 0.025 and 0.3 mM, we observed robust nematic bands along the trenches for intermediate spacings, *H* = 10–20 μm (Figure 6A). However, at large ATP concentrations, ≥ 0.3 mM, we observed polar flocks and nematic swarms that were uncorrelated with the etched features (Figure 6B; see also Supplementary Movie 7), with similar structure and dynamics as previous studies on planar substrates.^6^

Increasing the ATP concentration increases the rate of myosin attachment and detachment, as well as increases the number of myosin involved in pushing a single filament.^63^ We suspect that the additional tension imparted onto the filament makes it easier to escape the channels and facilitates “crowd-surfing” due to increased ATP-driven detachment. Similar flocking behavior is observed in the simulations when the active force on the filament is large (see the Supporting Information document).

**Figure 7:**
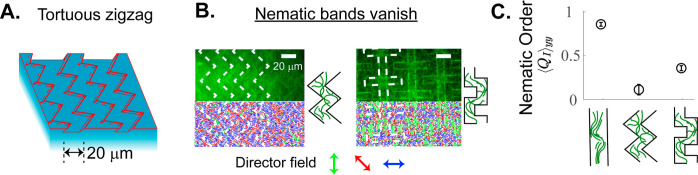
Etched substrates with tortuous topographies disrupt swarming nematic bands. (A) We designed etched substrates with tortuous zigzag patterns with *H* = 20 μm spacing. (B) Swarming nematic bands vanish on substrates with triangle- and square-wave patterns. Scale bars are 20 μm. (C) Comparing the *yy* component of the intensity-weighted nematic order tensor confirms the reduction of ordering along the *y*-direction on tortuous trenches compared to parallel trenches. All experiments are conducted at 0.05 mM ATP concentration.

Additionally, we studied the effect of in-plane tortuosity on nematic band formation by creating etched patterns that zigzag along the substrate. We hypothesized that the competition between the tortuosity path length and the swarm persistence length governs the onset of the nematic band formation. To test this hypothesis, we studied two zigzag patterns, with periodic triangle- and square-wave features, at a fixed spacing *H* = 20 μm (Figure 7). In both tortuous patterns, we observed accumulation of actin filaments along the boundaries but no swarming nematic bands. Tortuosity changes the preferred orientation at the channel edges, causing destructive interference between nematic boundary layers at various points along the surface. The nematic bands disappear when the tortuosity path length is comparable to the swarm persistence length. Although the biased orientation of an individual filament from one boundary can interact with other filaments on the opposing boundary, the tortuous path causes destructive interference with no collective enhancement of nematic ordering. This effect is similar to a spherical active particle moving through a porous media with a tortuous path, which is known to create density and polar boundary layers that can overlap destructively.^64,65^

The fact that directed swarms emerge when the topographic and filament length scales are commensurate suggests a type of coherence between collections of filaments in a corrugated landscape. In dense 2D systems, passive filaments with infinite persistence length (hard rods) are known to undergo a Berezinskii–Kosterlitz–Thouless (BKT) phase transition.^66,67^ In an unbounded system, the local orientation correlation decays algebraically over a length scale λ that depends upon the Frank elastic constant of a continuum nematic fluid.^68^ In our case, the soft walls bias the orientation of the filaments at the boundaries, and this propagates into the center of the channel over the distance λ, which is a function of the effective Frank elastic constant and the activity. The effective elastic constant depends on the physical properties of the filament (i.e., filament persistence length and contour length).^69,70^ We also vary the persistence length of the simulated filaments and show that the magnitude of nematic order increases and the location of the optimal spacing H* decreases as the persistence length grows (see the Supporting Information document). Scission events due to collisions create a large polydispersity in the actin contour length. This precludes us from meaningfully increasing the F-actin length to explore different ratios of the contour length and persistence length in experiments.

Consequently, nearby filaments align with the boundaries due to proximity to their neighbors. The finite concentration of filaments provides a mechanism of transmitting the nematic bias across multiple filaments up to a distance λ away from boundaries, beyond which orientational correlations between filaments rapidly fall off. If the corrugations are spaced less than λ, then nematic order persists throughout the entire channel.

**Figure 8:**
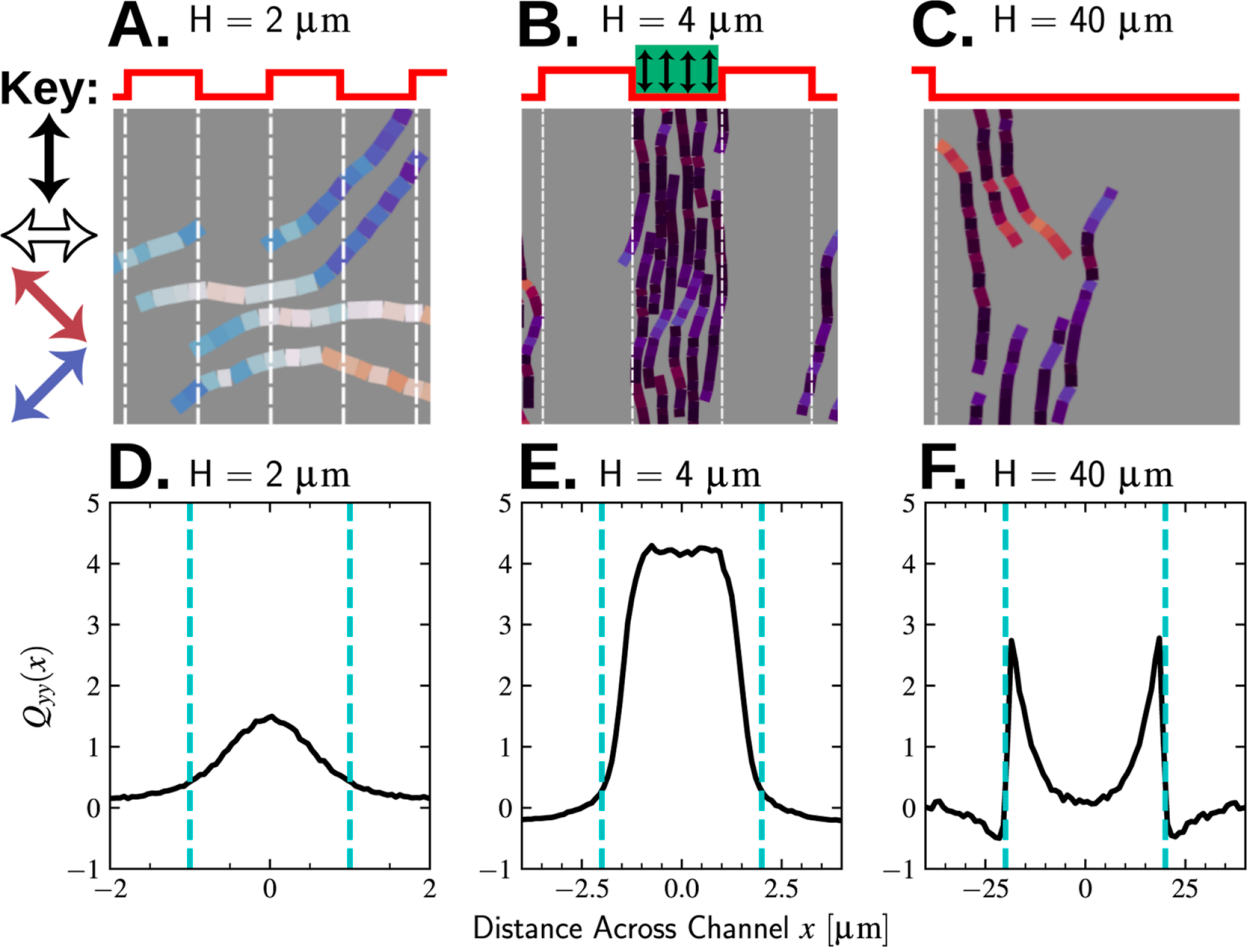
Nematic bands form when edge-induced nematic order is coherent through the entire channel. Top row (A–C) cropped simulation snapshots of filaments in a confining potential with different periodic repeat spacings. Filament segments are colored according to their director. White dashed lines indicate the edges of the potential wells where the gradient is strongest. Bottom row (D–F) local nematic order across a primitive periodic cell, obtained from eq 14. The origin of the *x*-axis is centered inside the potential well.

To help explain this idea of nematic coherence induced by an aligning bias, Figure 8A–C depicts simulation snapshots of a system of filaments with three different periodic repeat spacings, *H* = 2, 4, and 40 μm. In Figure 8D–F, we plotted histograms of the *yy* component of the *local* nematic order tensor, **Q**(*x*), corresponding to these snapshots. The local nematic order tensor is defined by “binning” filaments into a primitive section of the periodic potential landscape, spanning a distance – *H* ≤ *x* ≤ *H*
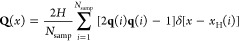
14where *x*_H_(*i*) = *H* + mod[*x*(*i*) + *H*, 2*H*] is the global displacement of the *i*th filament from the primitive periodic cell. In numerically evaluating eq 14, the Dirac delta function is approximated by a finite impulse function whose width and reciprocal height are equal to the bin size. Averaging eq 14 over the periodic interval, – *H* ≤ *x* ≤ *H* recovers the *average* nematic order tensor defined by eq 13
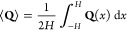
15therefore, the components of **Q**(*x*) are normalized such that their average over space ⟨**Q**⟩ has components bounded between ±1.

The snapshots and histograms presented in Figure 8 can be understood as follows. For the narrow spacing shown (*H* = 2 μm, Figure 8A,D), filaments are only partially biased by the potential gradient to align along the *y*-direction. This weak bias results from the fact that the filaments are much longer than the repeat spacing (*L*_c_ ≫ *H*, where *L*_c_ = 10 μm) and so frequently bridge multiple periodic cells under zero force and torque. Importantly, the peak nematic order is centered within the potential well. As *H* is increased to 4 μm (Figure 8B,E), the peak nematic order increases in scale and widens in extent to span nearly the entire well. This case corresponds to the “optimal” spacing, H*, as discussed in Figure 5. At much larger spacing (*H* = 40 μm, Figure 8C,F), the peak nematic order localizes near the edges of the potential well, where the gradient is strongest, and decays toward the center. The decay length (or coherence length), λ, is on the order of 10 μm and comparable to the filament contour length, *L*_c_. In this case, the size of the well is larger than the scale over which nematic order can be transmitted via interactions with other filaments, resulting in loss of nematic order across a “boundary layer” adjacent to the edges of the well. Comparing this case to the intermediate spacing (*H* = 4 μm) indicates that the highest order is achieved when the two boundary layers on either side of the well overlap.

## 4 Conclusions

Our experimental results demonstrate that surface topography can be employed to induce and control the ordering and swarming transitions of self-propelled filaments at finite surface density. We successfully modeled this coupling between topography and collective motion by using a 2D soft confinement potential, which reflects the local effect of bending filaments across the trench boundaries. The 2D system serves as a compelling model for understanding active force generation on curved surfaces of practical interest including 2D active materials and biological cell membranes. Whether this concept extends to 3D active fluids remains to be demonstrated but is worthy of further investigation.

Our findings highlight the intricate interplay between active propulsion, many–body interactions, and soft confinement, which orchestrate the emergence of ordered, regular patterns in active filaments under specific surface topographies. This observation suggests that the optimal confinement length scale, corresponding to the coherence length in active nematic systems, offers a valuable design principle for manipulating two-dimensional (2D) active fluids in confined geometries.

Future work in this project can focus on the utilization of collections of active filaments to enhance the capabilities of existing lab-on-a-chip devices. Specific topographical patterning can be used to locally enhance or repress the swarming of filaments, allowing for precise control of the location and direction of nematic bands and polar flocks. Additionally, incorporating tortuous channels offers a promising approach for sorting or separating incoming groups of filaments, facilitating tasks such as analyte detection while enabling greater filament surface density.

## Supporting Information Available

Additional simulation results demonstrating the impact of filament flexibility, active force, and tortuosity on the formation of nematic bands (PDF)
Experiment showing F-actin gliding on etched trenches with spacing *H* = 20 μm (robust nematic swarms) (MP4)
Experiment showing F-actin gliding on etched trenches with spacing *H* = 5 μm (no swarming observed) (MP4)
Experiment showing F-actin gliding on etched trenches with spacing *H* = 40 μm (no swarming observed) (MP4)
Simulation showing semiflexible filaments in a 2D potential field with spacing *H* = 2 μm (swarms not correlating with potential landscape) (MP4)
Simulation showing semiflexible filaments in a 2D potential field with spacing *H* = 4 μm (swarms directed along the “valleys” of the potential landscape) (MP4)
Simulation showing semiflexible filaments in a 2D potential field with spacing *H* = 40 μm (swarms persisting over a distance smaller than the repeat spacing) (MP4)
Experiment showing the formation of polar flocks of F-actin triggered by higher ATP concentration (trench spacing *H* = 20 μm) (MP4)
