# Supplementary material for: Surface Topography Induces and Orients Nematic Swarms of Active Filaments: Considerations for Lab-On-A-Chip Devices
Source: ACS Appl Nano Mater. 2024 May 8;7(10):12142–52. doi: 10.1021/acsanm.4c02020 (PMC11129142; doi:10.1021/acsanm.4c02020)
Supplement: Supplementary file 1 — an4c02020_si_001.pdf [file an4c02020_si_001.pdf]

Supporting Information for Surface Topography Induces and Orients Nematic Swarms of Active Filaments: Considerations for Lab-On-A-Chip Devices

Joseph M. Barakat[*,†,‡], Kevin J. Modica[†,‡], Le Lu[†], Stephanie Anujarerat[†], Kyu Hwan Choi[†], and Sho C. Takatori[*,†]

[†]Department of Chemical Engineering, University of California, Santa Barbara, Santa Barbara, CA 93106 USA.

[‡] These authors contributed equally to this work.

[*]Correspondence: Sho C. Takatori; stakatori@ucsb.edu, Joseph M. Barakat; josephbarakat@ucsb.edu

1 Sensitivity of nematic order to self-propulsion force

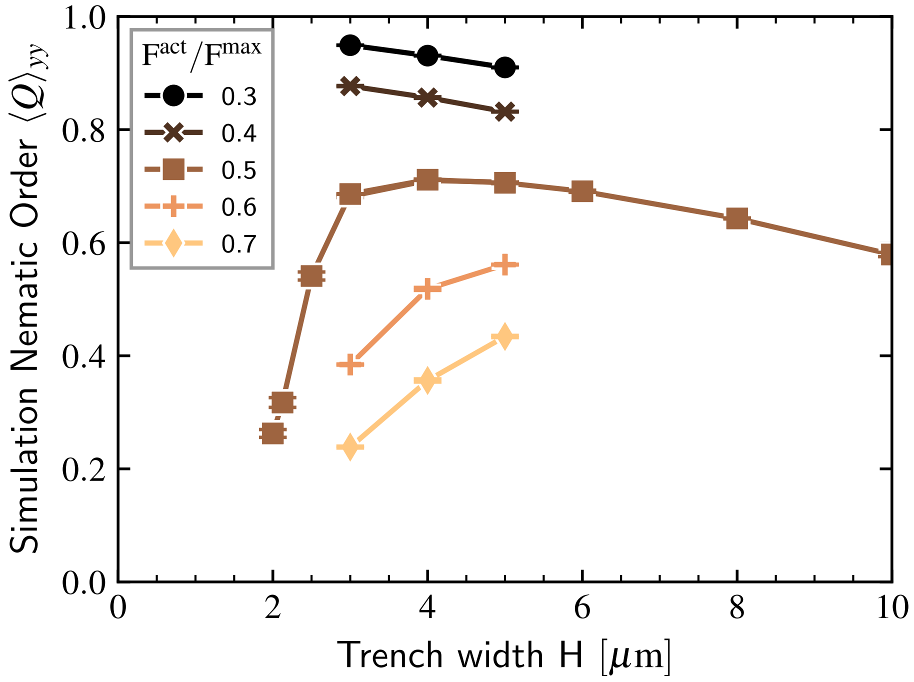

Figure S1: Simulations of active filaments with length 10 µm under different self-propulsive strengths. The global nematic order in the direction of the trench ($\langle Q \rangle_{yy}$) decreases as the activity increases in simulations of active filaments. The self-propulsive force (F^{act}) represents the force applied on each coarse-grained bead in the direction of the polymer tangent. The maximum restoring force (F^{max}) is imposed on the polymer by the gradient of the confining potential. As F^{act} increases relative to F^{max}, multiple beads pushing against the channel head-on perform a cooperative escape due to the greater combined force. This creates a increased capacity for "crowd-surfing" at high activity levels, which also increases the trench width corresponding to the maximum nematic order. All simulation parameters other than F^{act} are equivalent to those used in the main paper. Error bars are calculated by the standard deviation of the mean from 3 independent simulations and when not visible are smaller than the marker size.

2 Sensitivity of nematic order to filament persistence length

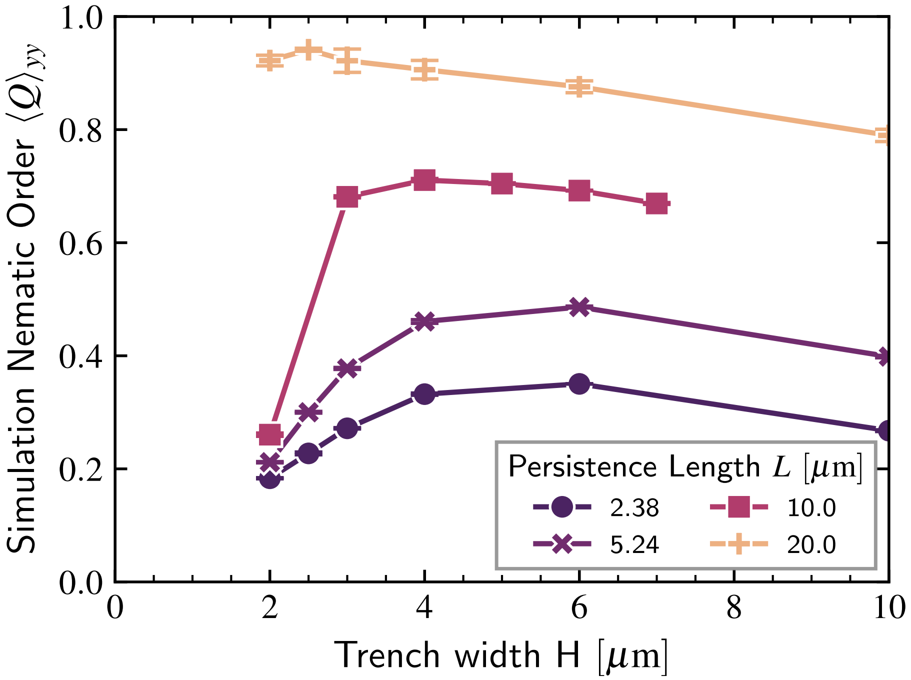

Figure S2: Simulations of active filaments with contour length L_c = 10 µm under different polymer persistence lengths (L). The global nematic order in the direction of the trench ($\langle Q \rangle_{yy}$) increases as the persistence length increases in simulations of active filaments. In simulation, flexible filaments are more able to reorient inside a channel to escape and begin "crowd-surfing". Additionally, more flexible filaments will have a lower nematic order due to the coiling of the polymer preventing alignment. The persistence length is defined by the ratio of the harmonic spring constant to bend relative to the thermal energy ($L = k_{\mathrm{ang}}/k_{\mathrm{B}}T$). All simulation parameters other than L are equivalent to those used in the main paper. Error bars are calculated by the standard deviation of the mean from 3 independent simulations.

3 Including tortuosity to the channel simulations

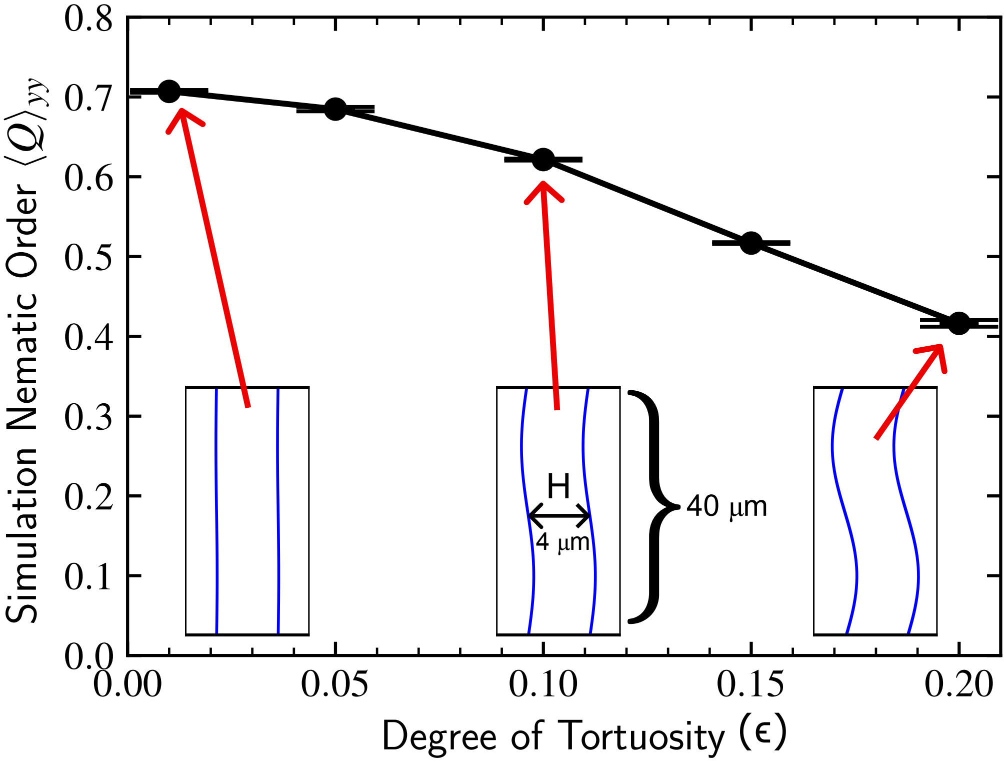

Figure S3: Simulations of active filaments in a tortuous channel. As the channels change from from straight to curved, the global nematic order in the direction of the trenches $\langle Q \rangle_{yy}$ decreases at constant trench width $H = 4$ µm. The curvature breaks up the formation and propagation of nematic swarms. The degree of tortuosity (ϵ) is defined in SI Eq. 1. All simulation parameters are equivalent to those used in the main paper. Error bars are calculated by the standard deviation of the mean from 3 independent simulations.

To simulate the properties of an active filament in a tortuous trench, we modify the external confining potential such that it varies in y as well as in x.

$$V_{\text{ext}}(\mathbf{r}_{i,j}) = \begin{cases} 0, & \text{if the } j\text{th filament crosses two or more ``edges,''} \\ -A \tanh\left[\frac{H}{\pi\sigma} \cos\left(\frac{\pi x}{H} + 2\epsilon\pi \sin\left[\frac{2\pi y}{\lambda_y}\right]\right)\right], & \text{otherwise.} \end{cases} \tag{1}$$

As in the main text, A is the amplitude of the potential, H is the trench width, σ is the diameter of the coarse-grained bead composing the filament. The argument of the cosine term has been modified from the main text to include variations in y. λ_y is the wavelength of the periodic channel bending, which shifts the potential energy minimum from $x = 0$ to $x = -2\epsilon\pi \sin[2\pi y/\lambda_y]$. We vary the "degree of tortuosity" ϵ from 0 (straight channel) to 0.2 to demonstrate how mild perturbations in the channel geometry affect the bulk nematic order.
